# Midazolam increases cisplatin-sensitivity in non-small cell lung cancer (NSCLC) via the miR-194-5p/HOOK3 axis

**DOI:** 10.1186/s12935-021-02104-6

**Published:** 2021-07-28

**Authors:** Tingting Sun, Jing Chen, Xuechao Sun, Guonian Wang

**Affiliations:** 1 Department of Anesthesiology, Harbin Medical University Cancer Hospital, Haping Road No. 150, Harbin, 150081 Heilongjiang China; 2 Institute of Pain, Harbin Medical University Sino-Russian Research Center, The Fourth Affiliated Hospital of Harbin Medical University, Yinhang Street No. 31, Harbin, 150001 Heilongjiang China

**Keywords:** Cisplatin-resistance, Midazolam, miR-194-5p, HOOK3, Non-small cell lung cancer

## Abstract

**Backgrounds:**

As previously reported, midazolam anesthesia exerts tumor-suppressing effects in non-small cell lung cancer (NSCLC), but the regulating effects of this drug on cisplatin-resistance in NSCLC have not been studied. Thus, we designed this study to investigate this issue and preliminarily delineate the potential molecular mechanisms.

**Methods:**

We performed MTT assay and trypan blue staining assay to measure cell proliferation and viability. Cell apoptosis was examined by FCM. qRT-PCR and immunoblotting were performed to determine the expression levels of genes. The targeting sites between genes were predicted by bioinformatics analysis and were validated by dual-luciferase reporter gene system assay. Mice tumor-bearing models were established and the tumorigenesis was evaluated by measuring tumor weight and volume. Immunohistochemistry (IHC) was used to examine the pro-proliferative Ki67 protein expressions in mice tumor tissues.

**Results:**

The cisplatin-resistant NSCLC (CR-NSCLC) cells were treated with high-dose cisplatin (50 μg/ml) and low-dose midazolam (10 μg/ml), and the results showed that midazolam suppressed cell proliferation and viability, and promoted cell apoptosis in cisplatin-treated CR-NSCLC cells. In addition, midazolam enhanced cisplatin-sensitivity in CR-NSCLC cell via modulating the miR-194-5p/hook microtubule-tethering protein 3 (HOOK3) axis. Specifically, midazolam upregulated miR-194-5p, but downregulated HOOK3 in the CR-NSCLC cells, and further results validated that miR-194-5p bound to the 3’ untranslated region (3’UTR) of HOOK3 mRNA for its inhibition. Also, midazolam downregulated HOOK3 in CR-NSCLC cells by upregulating miR-194-5p. Functional experiments validated that both miR-194-5p downregulation and HOOK3 upregulation abrogated the promoting effects of midazolam on cisplatin-sensitivity in CR-NSCLC cells.

**Conclusions:**

Taken together, this study found that midazolam anesthesia reduced cisplatin-resistance in CR-NSCLC cells by regulating the miR-194-5p/HOOK3 axis, implying that midazolam could be used as adjuvant drug for NSCLC treatment in clinical practices.

**Supplementary Information:**

The online version contains supplementary material available at 10.1186/s12935-021-02104-6.

## Background

Chemo-resistance in non-small cell lung cancer (NSCLC) is a huge obstacle that makes chemotherapy ineffective for NSCLC treatment, resulting in the worse prognosis and high morbidity for NSCLC patients worldwide, which seriously degrades the life quality of human beings [1–3]. Among all the chemical drugs, cisplatin is commonly used for NSCLC treatment and serves as the first-line chemical drug for NSCLC [4–6]. According to the clinical data, cisplatin is initially effective to kill NSCLC cells, however, as the results of continuous long-term cisplatin exposure-induced cisplatin-resistance, the NSCLC cells become resistant to further cisplatin stimulation [4–6]. Thus, it becomes urgent and necessary to develop novel strategies to improve cisplatin-sensitivity in the clinical practices. Thus, in recent studies, researchers concurrently focus on identifying novel cisplatin-resistance associated genes [7, 8] and searching for the adjuvant drugs which are capable of restoring cisplatin-sensitivity [9, 10]. Of note, the published data indicates that midazolam anesthesia can be used as potential anti-cancer drugs for hepatocellular carcinoma [11] and lung cancer [12], but no literatures report the involvement of midazolam in regulating chemo-resistance, especially in modulating cisplatin-sensitivity in NSCLC.

To our knowledge, investigations on uncovering the underlying mechanisms and identification of cancer-associated genes have been proved as effective treatment strategies to reverse chemo-resistance in NSCLC [7, 13, 14]. Among all types of the genes, MicroRNAs (miRNAs) are a class of small non-coding RNAs (ncRNAs) characterizing with post-transcriptional regulation activities [15–17], and multiple miRNAs involve in regulating cisplatin-resistance in NSCLC [7, 8, 18]. For example, Ma et al. find that miR-425-3p confers cisplatin-resistance in NSCLC [7], Lin et al. evidence that miR-140 re-sensitizes cisplatin-resistant NSCLC cells to cisplatin treatment [18], and Pan et al. notice that miR-138-5p modulates cisplatin-resistance in A549/DDP cells via suppressing ATG7-mediated autophagy [8]. Interestingly, midazolam is reported to suppress cancer progression in hepatocellular carcinoma via modulating miRNAs [11], indicating that midazolam may participate in the regulation of cisplatin-resistance in NSCLC via miRNAs. According to the data from our preliminary experiments, we screened out one of the cisplatin-resistance associated miRNA, miR-194-5p [19–21], that could be positively regulated by midazolam.

Hook microtubule-tethering protein 3 (HOOK3) is one of the homologues of HOOK family, which is abundantly enriched in human cells and functions as adaptor proteins to facilitate the trafficking of membranes among Golgi apparatus [22], centrosomes [23], endosomes [24] and lysosomes [24, 25], and high-levels of HOOK3 can be used as an independent predictor of poor prognosis in prostate cancer [26], but the role of HOOK3 in regulating cancer progression and drug resistance has not been studied. To our knowledge, miRNAs exert their biological functions through targeting the 3’ untranslated region (3’UTR) of their downstream target genes [27, 28], and specifically, multiple cancer associated genes, including FOXA1 [29], SLC40A1 [21], and IGF1R [30], can be targeted and degraded by miR-194-5p. Through performing the bioinformatics analysis, this study predicted that there existed targeting sites between miR-194-5p and 3’UTR of HOOK3, which indicted their potential regulating relationship.

In general, this study was designed to investigate the possible utilization of midazolam anesthesia to recover cisplatin-sensitivity for NSCLC treatment, and explored the involvement of the miR-194-5p/HOOK3 axis in modulating this process.

## Materials and methods

### Clinical specimens

The clinical NSCLC tissues (N = 38) were collected from patients with (N = 16) or without cisplatin-resistant (N = 22) properties, the criteria for the judgement of cisplatin-resistant characteristics was conducted by two experienced doctors in our hospital. After surgical resection, the tissues were immediately stored at −70 ℃ refrigerator for further analysis. The Ethics Committee Affiliated to Harbin medical university Cancer Hospital approved our clinical experiments, and the informed consent forms had been obtained from all the participants.

### Induction of CR-NSCLC cells

The CS-NSCLC cells (A549 and H1299) were purchased from ATCC (USA) and were cultured in the DMEM medium (Gibco, CA, USA) with 10% FBS (Gibco, USA) supplementation. Then, the cells were subjected to continuous low-dose cisplatin exposure (0–10 μg/ml, 45 days) to establish CR-NSCLC cells (A549/DDP and H1299/DDP) according to the experimental protocols provided by the previous publication [31], and the CR-NSCLC cells were maintained in the medium containing 1 μg/ml cisplatin. Finally, the CR-NSCLC cells were treated with high-dose cisplatin (50 μg/ml) and midazolam (10 μg/ml) for further analysis.

### Vector transfection

The miR-194-5p mimic and inhibitor, and HOOK3 overexpression vectors were synthesized by GenePharma (Suzhou, China) according to the previous publications, which were then transfected into the NSCLC cells by using the Lipofectamine transfection reagent (Invitrogen, CA, USA) in accordance with the manufacturer’s instruction. Vectors transfection efficiency was determined by Real-Time qPCR analysis.

### Real-Time qPCR

Real-Time qPCR was conducted to examine genes expression at mRNA levels according to the protocols provided by the previous work. Briefly, total RNA was extracted by TRIzol reagent (Invitrogen, CA, USA), reversely transcribed into cDNA, and quantified by using the Real-Time qPCR kit (TAKARA, Japan) according to the producer’s instructions. We designed the primers for miR-21 [32], miR-198 [33], miR-423-5p [34], miR-425-3p [35], miR-194-5p [29], miR-328 [36], miR-454-3p [37], miR-199a [38], HOOK3 [26], U6 [29], GAPDH [29] according to the sequences provided by the previous publications, which were synthesized by the commercial third-party company (Sangon Biotech, Shanghai, China).

### Western Blot analysis

The expression levels of HOOK3 protein were measured by performing Western Blot analysis as previously described [26]. In brief, RIPA lysis buffer was purchased for total protein extraction, which were then subjected to 10% SDS-PAGE for separation according to their molecular weight, and the target proteins were transferred onto the PVDF membranes (Millipore, USA) and were blocked by 5% skim milk for 2 h at room temperature. The membranes were sequentially probed with the primary antibodies against HOOK3 (1:2000, Abcam, UK) and GAPDH (1:1500, Abcam, UK) at 4 ℃ overnight, and the secondary antibodies (1:3000, Abcam, UK) for 1 h at room temperature. The proteins bands were finally visualized by the enhanced chemiluminescence (ECL) system (ThermoFisher Scientific, USA) and analyzed by using the Image J software.

### Examination of cell proliferation and viability

The NSCLC cells were cultured in the 96-well plates at the density of 1000 cells per well for 0 h, 6 h, 12 h, 24 h and 48 h, respectively. The MTT assay was perfoemed to determine cell proliferation abilities. Specifically, 10 μg of MTT solution was added to each well of the 96-well plates, and the cells were subsequently cultured for 4 h. Then, the original medium was discarded, and 150 μl of DMSO was added to resolve the formazan, the plates were fully vortexed and a microplate reader was employed to determine optical density (OD) values at the wavelength of 450 nm. Moreover, cell viability was evaluated by trypan blue staining assay, and the cells were stained with trypan blue staining dye for 20 min at 37 ℃, a light microscope was used to count the dead blue cells, which was used to indicate relative cell viability.

### Apoptosis detection kit

As previously described [11], the NSCLC cells were fixed by using 4% paraformaldehyde for 10 min at room temperature, which were then respectively stained with Annexin V-FITC and PI, and a flow cytometer (FCM, BD Bioscience, USA) was employed to determine the Annexin V-FITC or PI-positive apoptotic cell ratio.

### In vivo animal experiments

Nine BALB/c nude mice (male, aged 6 weeks) were obtained from the animal center of Harbin Medical University, and were fed in the specific-pathogen-free (SPF) environment. The A549/DDP cells were subcutaneously injected into the dorsal flank of the mice with the concentrations of 2 × 10^7^ cells per mouse. The tumor formation, growth and volume were monitored every 5 days from day 0 to day 25. At day 25 post-injection, the mice were anesthetized and sacrificed, and the tumors were obtained and weighed. In addition, the mice tumor tissues were stored for further analysis.

### Evaluation of ki67 protein levels in mice tissues

The mice tumor tissues were prepared and embedded into the paraffin, and were subsequently spliced into sections with 5 μm thickness. The samples were de-waxed, and the antigens were repaired. Then, the tissues were sequentially blocked with hydrogen peroxide (10–15 min), incubated with the primary antibody against Ki67 (1:2000, Abcam, UK) for 20 min at room temperature and the HRP Polymer labelled secondary antibodies (Abcam, UK) for 30 min at room temperature without light exposure. Next, the samples were added with DAB solution and the incubation sustained for 3–15 min, and a light microscope (ThermoFisher Scientific, USA) was used to photograph the images, and the yellow cells were regarded as Ki67-positive cells. The IHC images were analyzed by Image J software, and the expression status of Ki67 protein was indicated by H-score.

### Dual-luciferase reporter gene system assay

The online starBase Software (http://starbase.sysu.edu.cn/) was performed to predict the targeting sites in miR-194-5p and 3’UTR of HOOK3 mRNA, and the binding sites in wild-type HOOK3 (Wt-HOOK3) were mutated and named as mutant HOOK3 (Mut-HOOK3). The above sequences were respectively incorporated into the luciferase reporter vectors, and co-transfected with the miR-194-5p mimic into the A549 and H1299 cells, and a Dual-luciferase reporter gene system (ThermoFihser Scientific, USA) was employed to quantify the relative luciferase activities in the above cells.

### Data collection and analysis

The SPSS 11.0 software was employed to analyze the data, which were presented as means ± standard deviation (SD). Statistical analysis was performed by the Student’s t-test for two groups, and one-way ANOVA for multiple groups (> 2), respectively. Correlation of differential gene expressions was analyzed by Pearson Correlation analysis. *P < 0.05, ** *P* < 0.01 and *** *P* < 0.001 were respectively indicated. Single experiment contained at least 3 repetitions.

## Results

### Establishment of CR-NSCLC cells by continuous low-dose cisplatin exposure

**Fig. 1:**
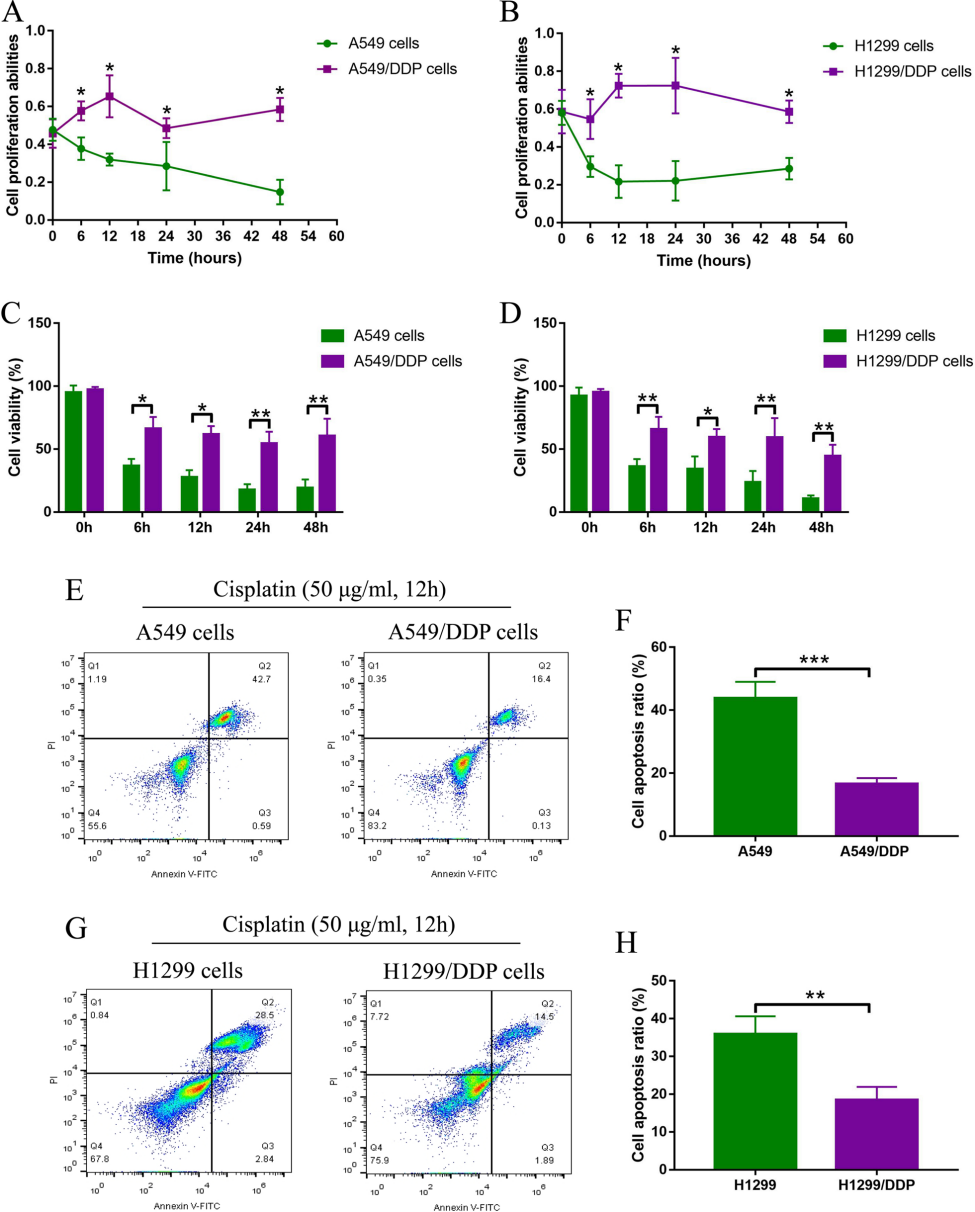
CR-NSCLC cells were successfully inducted from the parental CS-NSCLC cells. The CS-NSCLC and CR-NSCLC cells were exposed to high-dose cisplatin treatment at differential time points, and **A**, **B** cell proliferation abilities and **C**, **D** cell viability were respectively examined by MTT assay and trypan blue staining assay. **E**–**H** The NSCLC cells were subjected to high-dose cisplatin treatment for 24 h, and the Annexin V-FITC or PI-positive apoptotic cells were examined by performing the FCM analysis. Individual experiment contained at least 3 repetitions, and *P < 0.05, **P < 0.01, and ***P < 0.001 was considered as statistical significance

The CR-NSCLC cells (A549/DDP and H1299/DDP) were established from the parental CS-NSCLC (A549 and H1299) by using the continuous low-dose cisplatin exposure methods as previously described [31], and our data suggested that cisplatin brought the inhibitory effects on NSCLC cells in a dose-dependent manner (Additional file 1: Figure S1A, C), and the IC50 values were higher in CR-NSCLC cells (58 ± 4 μg/ml and 69 ± 2 μg/ml) than that of CS-NSCLC cells (18 ± 3 μg/ml and 16 ± 2 μg/ml) (Additional file 1: Figure S1B, D). Based on the above information, we selected high-dose cisplatin (50 μg/ml) to treat NSCLC cells in the following experiments, which was effective to kill CS-NSCLC cells, but its inhibiting effects on CR-NSCLC cell viability were not significant. The above cells were then subjected to high-dose cisplatin (50 μg/ml) treatment for 0 h, 6 h, 12 h, 24 h and 48 h. As shown in Fig. 1A, B, the MTT assay results showed that high-dose cisplatin significantly inhibited cell proliferation in CS-NSCLC cells in a time-dependent manner, but had little effects on the CR-NSCLC cells, which were supported by the following trypan blue staining assay results that high-dose cisplatin especially suppressed cell viability in CS-NSCLC cells (Fig. 1C, D). Next, the FCM assay was performed to examine cell apoptosis ratio, and we expectedly found that high-dose cisplatin significantly promoted cell apoptosis in the CS-NSCLC cells, comparing to the descendent CR-NSCLC cells (Fig. 1E–H), suggesting that the CR-NSCLC cells were successfully established in this study.

### Midazolam increased cisplatin-sensitivity in CR-NSCLC cells in vitro and in vivo

**Fig. 2:**
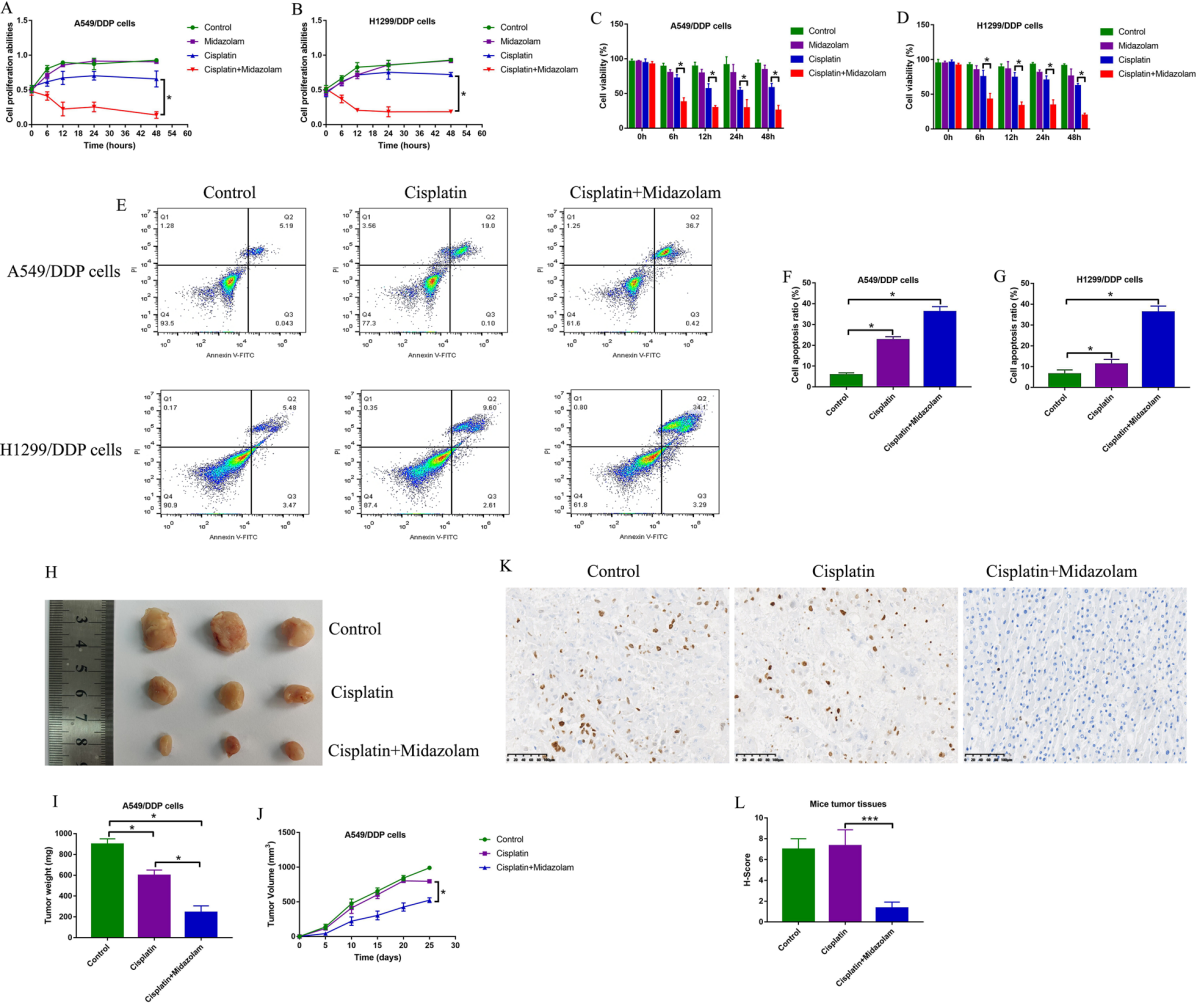
Midazolam enhanced cisplatin’s cytotoxic effects on CR-NSCLC cells. The CR-NSCLC cells were respectively treated with cisplatin and midazolam for 0 h, 6 h, 12 h, 24 h and 48 h, and **A**, **B** MTT assay and **C**, **D** trypan blue staining assay were used to measure cell proliferation and viability. **E**–**G** At 24 h post-treatment, cell apoptosis ratio was evaluated by using the FCM assay. The xenograft tumor-bearing mice models were established by using the A549/DDP cells, and **H**, **I** tumor weight and (J) volume were monitored to evaluate tumor growth in vivo. **K**, **L** The mice tumor tissues were collected and prepared, and the expression status of pro-proliferation Ki67 protein was determined by IHC, which were quantified by using the H-score. Individual experiment contained at least 3 repetitions, and *P < 0.05, **P < 0.01, and ***P < 0.001 was considered as statistical significance

Based on our preliminary experiments (Additional file 2: Figure S2A–D), midazolam suppressed cell viability in both CS-NSCLC cells and CR-NSCLC cells in a concentration-dependent manner, and the IC50 value for CS-NSCLC cell (60 ± 8 μg/ml and 55 ± 4 μg/ml) and CR-NSCLC cells (58 ± 4 μg/ml and 53 ± 7 μg/ml) were respectively shown. Of note, there existed no statistical significance between CS-NSCLC cells and CR-NSCLC cells regarding to IC values for midazolam. Given that low-dose midazolam (10 μg/ml) had little inhibiting effects on cell viability in NSCLC cells (Additional file 2: Figure S2A–D), we selected this concentration of midazolam for further investigations. Then, the CR-NSCLC cells (A549/DDP and H1299/DDP) were exposed to low-dose midazolam (10 μg/ml) and high-dose cisplatin (50 μg/ml) treatments, and the cells were divided into groups as follows: Control, midazolam alone group, cisplatin alone group, and cisplatin plus midazolam group. The MTT assay (Fig. 2A, B) and trypan blue staining assay (Fig. 2C, D) results showed that both midazolam alone and cisplatin alone had little effects on cell proliferation and viability in CR-NSCLC cells, while midazolam plus cisplatin significantly promoted CR-NSCLC cell death (Fig. 2A–D). In addition, the above results were supported by the following FCM assay results, which showed that midazolam increased cell apoptosis ratio in the high-dose cisplatin treated CR-NSCLC cells (Fig. 2E, F). Moreover, the xenograft tumor-bearing mice models were established by using the A549/DDP cells, which were then subjected to midazolam and cisplatin treatments, and the data in Additional file 3: Figure S3 showed that the above treatments did not influence mice body weight before surgical resection. The results verified that midazolam enhanced the inhibiting effects of cisplatin on tumor growth and tumorigenesis in vivo (Fig. 2H–J). In addition, Ki67 is identified as an important pro-proliferative protein facilitating the development of cancers [39], and by conducting immunohistochemistry (IHC) assay analysis, we found that midazolam plus cisplatin decreased Ki67 protein levels in mice tumor tissues (Fig. 2K, L). Those data suggested that midazolam was capable of increasing cisplatin-sensitivity in CR-NSCLC cells.

### Silence of miR-194-5p rescued cell viability in midazolam and cisplatin co-treated CR-NSCLC cells

**Fig. 3:**
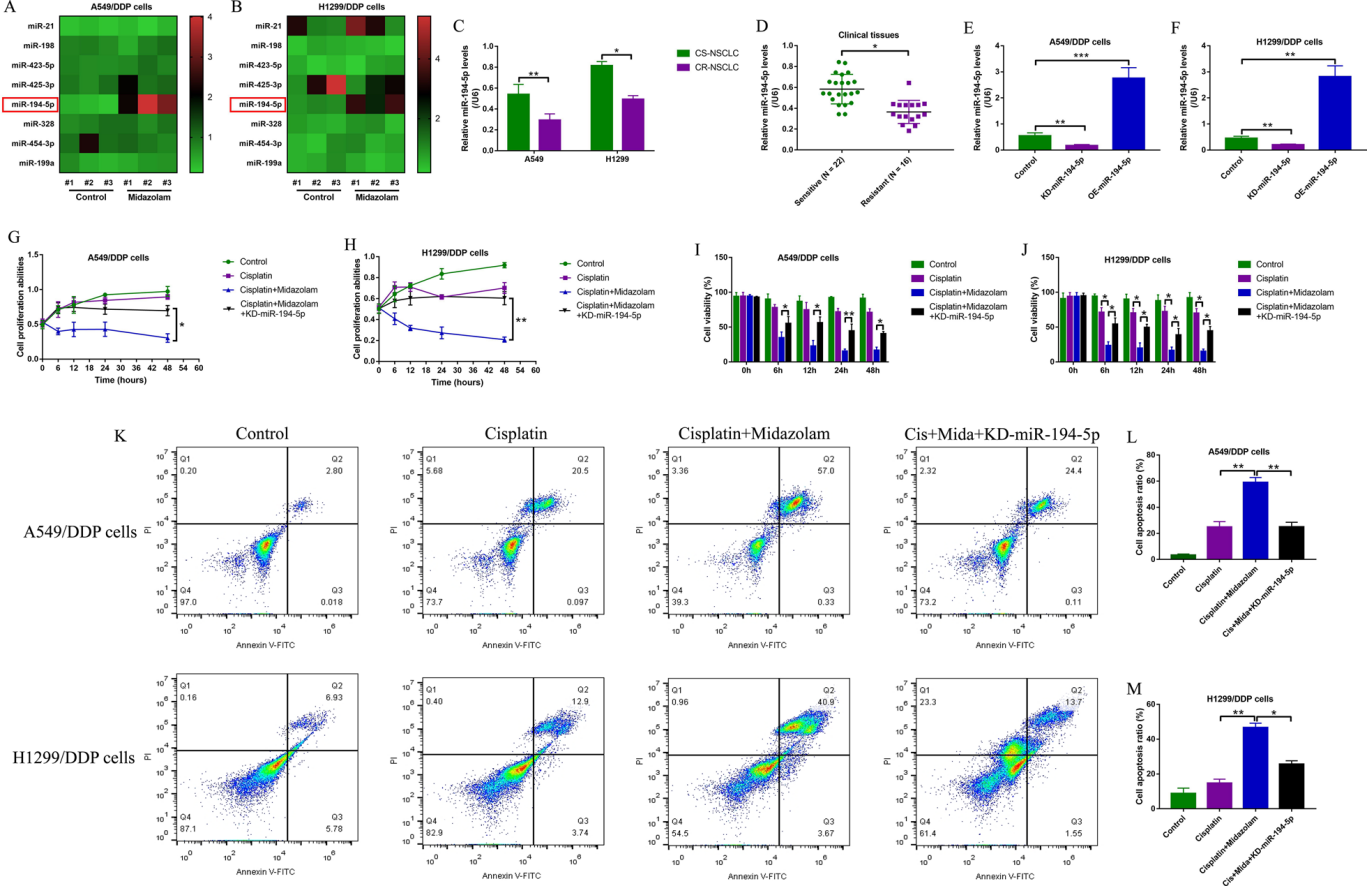
Midazolam upregulated miR-194-5p to improve cisplatin-sensitivity in CR-NSCLC cells. **A**, **B** The A549/DDP cells and H1299/DDP cells were stimulated with midazolam for 24 h, and Real-Time qPCR was performed to examine the expression levels of the eight cisplatin-resistance associated miRNAs, which were shown by using the heat map. The expression levels of miR-194-5p in CS-NSCLC and CR-NSCLC **C** cells and **D** clinical tissues were measured by performing the Real-Time qPCR analysis. **E**, **F** The miR-194-5p mimic and inhibitor were respectively transfected into the CR-NSCLC cells, and the transfection efficiency was determined. **G**, **H** MTT assay and (I, J) trypan blue staining assay were conducted to investigate the effects of cisplatin, midazolam and miR-194-5p inhibitor on cell proliferation and viability. **K**–**M** Cell apoptosis in the A549/DDP and H1299/DDP cells was determined by FCM. Individual experiment contained at least 3 repetitions, and *P < 0.05, **P < 0.01, and ***P < 0.001 was considered as statistical significance

MicroRNAs (miRNAs) are a class of small non-coding RNAs that are closely associated with cisplatin-resistance in NSCLC [7] and can be modulated by midazolam [11], which convinced us to investigate whether midazolam regulated cisplatin-resistance in NSCLC by modulating miRNAs. Thus, eight cisplatin-resistance associated miRNAs (miR-21, miR-198, miR-423-5p, miR-425-3p, miR-194-5p, miR-328, miR-454-3p and miR-199a) were selected and screened by performing Real-Time qPCR in this study, and we surprisingly found that midazolam specifically upregulated miR-194-5p, instead of other miRNAs, in both A549/DDP cells (Fig. 3A) and H1299/DDP cells (Fig. 3B). Further experiments evidenced that miR-194-5p was low-expressed in both CR-NSCLC cells (Fig. 3C) and clinical tissues (Fig. 3D), in contrast with their CS-NSCLC counterparts. The above data encouraged us to speculate that midazolam might regulate cisplatin-resistance in NSCLC via upregulating miR-194-5p. To validate this hypothesis, miR-194-5p inhibitor was transfected into the CR-NSCLC cells for its downregulation (Fig. 3E, F), and the following MTT assay (Fig. 3G, H) and trypan blue staining assay (Fig. 3I, J) results evidenced that the inhibiting effects of midazolam on cell proliferation and viability in cisplatin-treated CR-NSCLC cells were abrogated by silencing miR-194-5p. Moreover, knockdown of miR-194-5p suppressed midazolam plus cisplatin-induced cell apoptosis in CR-NSCLC cells (Fig. 3K–M), hinting that midazolam increased cisplatin-sensitivity in CR-NSCLC cells in a miR-194-5p-dependent manner.

### Midazolam negatively regulated HOOK3 by inducing miR-194-5p upregulation

**Fig. 4:**
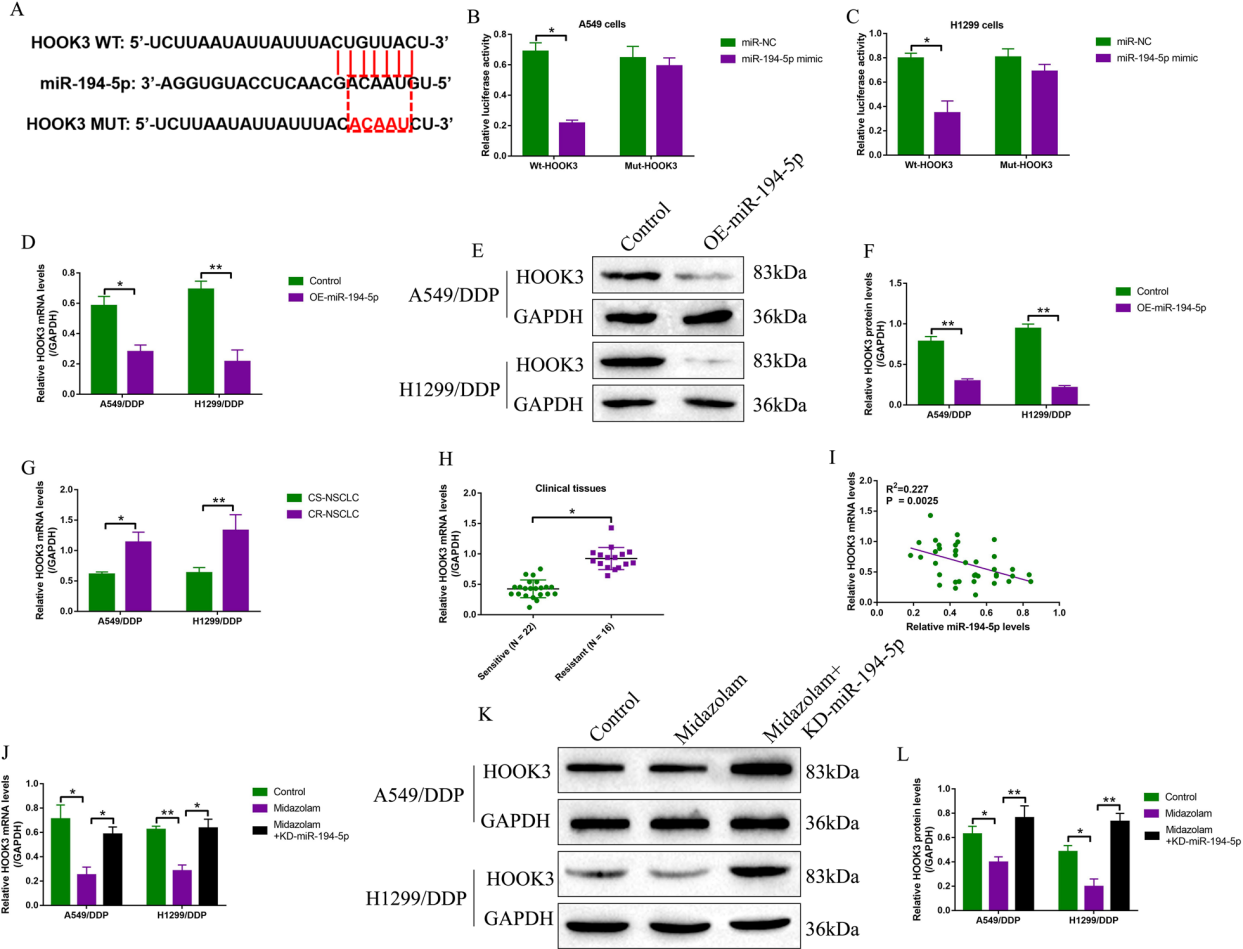
Midazolam negatively regulated HOOK3 via upregulating miR-194-5p. **A** The targeting sites between miR-194-5p and 3’UTR of HOOK3 were predicted by our bioinformatics analysis, and the bindings sites in HOOK3 were mutated. **B**, **C** The dual-luciferase reporter gene system assay was conducted to validate the targeting sites in miR-194-5p and HOOK3. Upregulation of miR-194-5p suppressed HOOK3 expressions at both **D** mRNA and **E**, **F** protein levels, as determined by Real-Time qPCR and Western Blot analysis. Real-Time qPCR analysis elucidated that HOOK3 mRNA tended to be enriched in CR-NSCLC **G** cells and **H** clinical tissues. **I** MiR-194-5p and HOOK3 mRNA were negatively correlated in the clinical NSCLC tissues, as determined by Pearson correlation analysis. **J**–**L** Knock-down of miR-194-5p rescued HOOK3 expressions in the cisplatin and midazolam co-treated CR-NSCLC cells. Individual experiment contained at least 3 repetitions, and *P < 0.05, **P < 0.01, and ***P < 0.001 was considered as statistical significance

As previously reported [27, 28], miRNAs usually target the 3’ untranslated region (3’UTR) of the downstream cancer associated genes to participate in the regulation of cancer biology and drug-resistance, and by performing the online starBase software, we identified that 3’UTR of HOOK3 mRNA could be targeted by miR-194-5p (Fig. 4A), and the following dual-luciferase reporter gene system assay results evidenced that miR-194-5p mimic specifically suppressed luciferase activities in the CS-NSCLC cells co-transfected with wild-type HOOK3 luciferase vectors (Fig. 4B, C). Further Real-Time qPCR (Fig. 4D) and Western Blot analysis (Fig. 4E, F) results validated that miR-194-5p negatively regulated HOOK3 at both mRNA and protein levels in the A549/DDP cells and H1299/DDP cells. Then, we noticed that HOOK3 was significantly upregulated in the CR-NSCLC cells (Fig. 4G) and clinical tissues (Fig. 4H), comparing to the CS-NSCLC cells and tissues, and the Pearson correlation analysis results supported that miR-194-5p negatively correlated with HOOK3 mRNA in the NSCLC tissues (Fig. 4I). Moreover, we evidenced that midazolam suppressed HOOK3 expressions in CR-NSCLC cells, which were rescued by silencing miR-194-5p (Fig. 4J–L), implying that midazolam negatively regulated HOOK3 through upregulating miR-194-5p in CR-NSCLC cells.

### Upregulation of HOOK3 abrogated the promoting effects of midazolam on cisplatin-sensitivity in CR-NSCLC cells

**Fig. 5:**
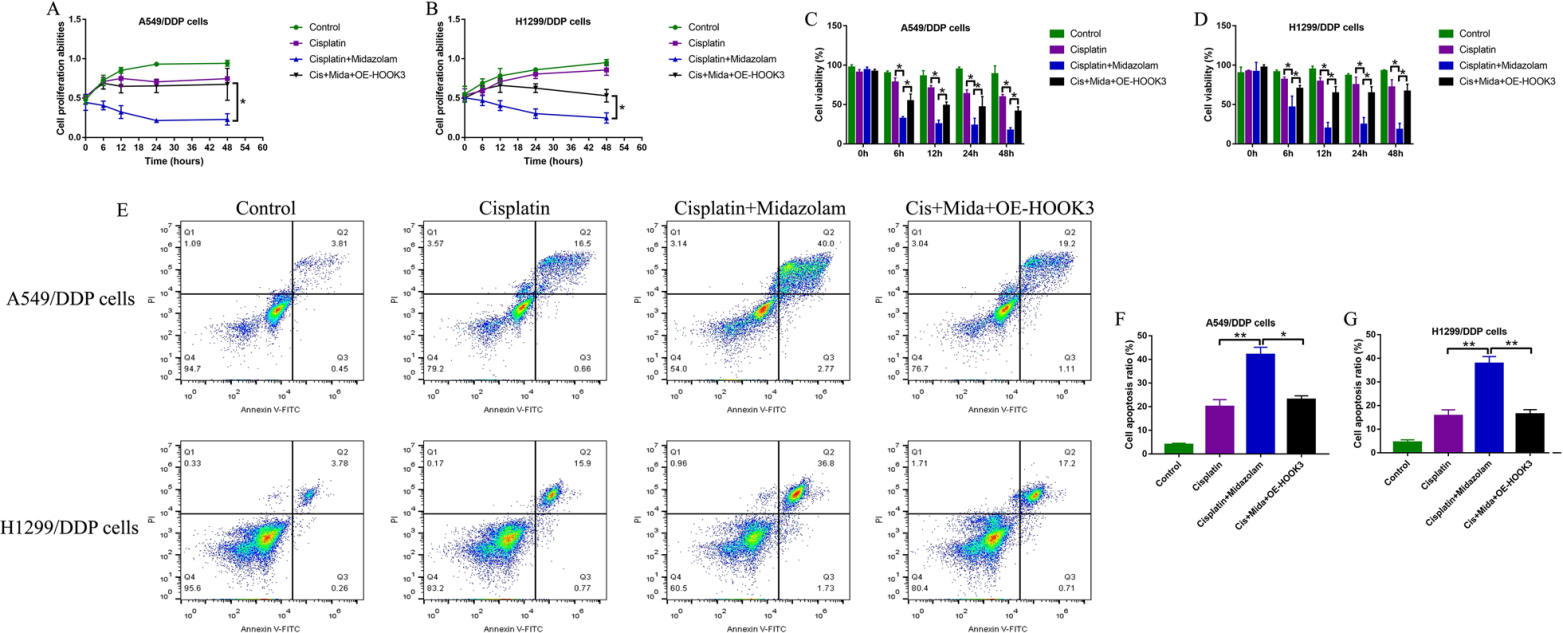
Upregulation of HOOK3 rescued cell viability in midazolam and cisplatin co-treated CR-NSCLC cells. We performed **A**, **B** MTT assay and **C**, **D** trypan blue staining assay to respectively examine cell proliferation and viability in the CR-NSCLC cells. **E**–**G** Cell apoptosis ratio in CR-NSCLC cells was examined by FCM assay. Individual experiment contained at least 3 repetitions, and *P < 0.05, **P < 0.01, and ***P < 0.001 was considered as statistical significance

Given that midazolam inhibits HOOK3 in NSCLC cells, and HOOK3 is closely associated with cancer progression [26], it was reasonable to hypothesize that midazolam might regulate cisplatin-resistance in NSCLC via HOOK3. To validate this speculation, the CR-NSCLC cells were respectively treated with midazolam, high-dose cisplatin, and HOOK3 overexpression (OE-HOOK3) vectors. The MTT assay results showed that the inhibiting effects of midazolam plus cisplatin co-treatments on cell proliferation were abrogated by upregulating HOOK3 (Fig. 5A, B), which were supported by the following trypan blue staining assay results that HOOK3 overexpression rescued cell viability in the CR-NSCLC cells co-treated with midazolam and cisplatin (Fig. 5C, D). In addition, midazolam promoted cell apoptosis in cisplatin treated CR-NSCLC cells, which were also reversed by upregulating HOOK3, as examined by FCM assay in Fig. 5E–G. The above data indicated that midazolam increased cisplatin-sensitivity in CR-NSCLC cells through downregulating HOOK3.

## Discussion

Cisplatin-resistance makes this chemical drug ineffective for NSCLC treatment, resulting in worse prognosis and high-mortality in NSCLC patients after chemo-therapy, which brings huge health burden for human beings world [1–3]. Nowadays, researchers concentrate on identifying novel cancer-associated genes [7, 8] and developing adjuvant drugs [9, 10] to improve cisplatin-sensitivity to recover cisplatin-sensitivity and rescue the therapeutic efficacy of cisplatin. Especially, the potential therapeutic agents, such as Chinese medicine [40] and anesthetic agents [11, 12, 41–44], can eliminate chemo-resistance to some extent. For example, the commonly used propofol anesthesia is verified to recover trastuzumab-sensitivity in breast cancer [44], increase cisplatin-sensitivity in lung cancer [42] and ovarian cancer [43], and suppress Docetaxel-resistance in prostate cancer [41]. As one of the anesthesia, midazolam has also been used as anti-cancer drug for hepatocellular carcinoma [11] and lung cancer [12], but it is still unknown whether midazolam can be used as alternative drug to improve cisplatin-sensitivity in NSCLC. In this study, the CR-NSCLC cells were established as previously described [31], and we proved that midazolam significantly suppressed cell viability and increased cell apoptosis ratio in cisplatin treated CR-NSCLC cells, indicating that midazolam was effective to reverse cisplatin-resistance properties in CR-NSCLC cells, which were partially supported by the previous literatures [41–44].

As the post-transcriptional regulators, miRNAs are closely associated with cancer progression [45, 46] and cisplatin-resistance [7, 8, 18] in NSCLC, and targeting miRNAs has been proved as an effective strategy to recover cisplatin-sensitivity in NSCLC [7, 8, 18]. In addition, the expression levels of miRNAs can be modulated by midazolam [11], which convinced us that midazolam might regulate cisplatin-resistance in NSCLC cells via modulating miRNAs. To validate this hypothesis, Real-Time qPCR was used to profile the cisplatin-resistance associated miRNAs, and we surprisingly found that miR-194-5p but not other miRNAs could be positively regulated by midazolam in CR-NSCLC cells, and miR-194-5p was significantly downregulated in CR-NSCLC cells and tissues, in contrast with their cisplatin-sensitive counterparts, which were supported by the previous publications that miR-194-5p increases cisplatin-sensitivity in NSCLC [19–21]. Next, we evidenced that midazolam recovered cisplatin-sensitivity in CR-NSCLC cells via upregulating miR-194-5p. Specifically, the inhibiting effects of midazolam on cell viability in cisplatin-treated CR-NSCLC cells were abrogated by silencing miR-194-5p.

Given that miR-194-5p exerts its biological functions through targeting the 3’UTR of its downstream target genes for their inhibition and degradation [21, 29, 30], we screened out and verified that HOOK3 could be targeted and negatively regulated by miR-194-5p in NSCLC cells. Furthermore, we noticed that the inhibiting effects of midazolam treatment on HOOK3 expressions were reversed by downregulating miR-194-5p, suggesting that midazolam suppressed HOOK3 by upregulating miR-194-5p, which were partially supported by the previous study that midazolam regulated mRNA expressions via modulating miRNAs [11]. Moreover, as previously described, high-levels of HOOK3 predicts worse prognosis in prostate cancer [26], but it is still unclear the mechanistic role of HOOK3 in regulating cancer progression and drug resistance. Then, by performing the following gain-of-function experiments, we found that the rescuing effects of midazolam on cisplatin-sensitivity in CR-NSCLC cells were abolished by upregulating HOOK3, suggesting that midazolam downregulated HOOK3 to increase cisplatin-sensitivity in NSCLC, which indirectly reflected that HOOK3 participated in the regulation of drug resistance in NSCLC.

## Conclusions

Thus, we summarized the significances of this study as follows: (1) We firstly evidenced that midazolam could be used as adjuvant drug to improve cisplatin-sensitivity in NSCLC. (2) Midazolam exerted its biological effects by regulating the miR-194-5p/HOOK3 axis. Although the effects and underlying mechanisms by which midazolam enhanced cisplatin-sensitivity in NSCLC had been preliminarily investigated in vitro and in vivo, the conclusions presented in the present study were still needed to be validated by performing further clinical experiments.

## Supplementary Information

**Additional file 1: Figure S1.** (A, C) The inhibitory effects of differential doses of cisplatin on NSCLC cells. (B, D) The IC50 values for cisplatin in CS-NSCLC cells and CR-NSCLC cells were shown. Each experiment repeated at least 3 times, and *P < 0.05 and **P < 0.01 were considered as statistical significance.

**Additional file 2: Figure S2.** (A, C) The inhibitory effects of differential doses of midazolam on NSCLC cells. (B, D) The IC50 values for midazolam in CS-NSCLC cells and CR-NSCLC cells were shown. Each experiment repeated at least 3 times, and *P < 0.05 and **P < 0.01 were considered as statistical significance.

**Additional file 3: Figure S3.** The mice body weights were measured before surgical resection. Each experiment repeated at least 3 times.

## Data Availability

We had included all the data and materials in the final version of the manuscript.
