# Supplementary figures and images for: Midazolam increases cisplatin-sensitivity in non-small cell lung cancer (NSCLC) via the miR-194-5p/HOOK3 axis
Source: Cancer Cell Int. 2021 Jul 28;21:401. doi: 10.1186/s12935-021-02104-6 (PMC8317376; doi:10.1186/s12935-021-02104-6)

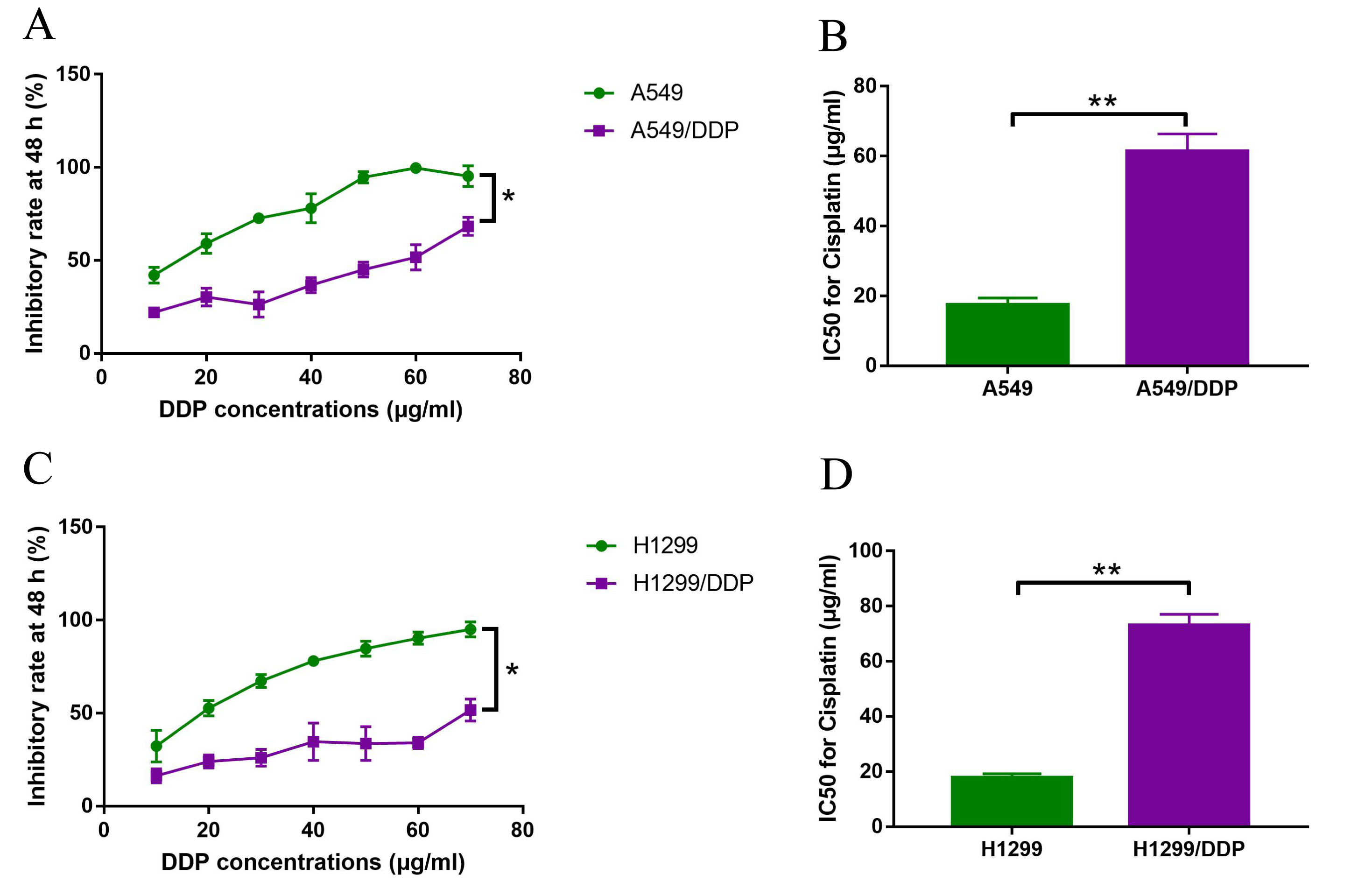

Supplement: Supplementary file 1 — Additional file 1: Figure S1. (A, C) The inhibitory effects of differential doses of cisplatin on NSCLC cells. (B, D) The IC50 values for cisplatin in CS-NSCLC cells and CR-NSCLC cells were shown. Each experiment repeated at least 3 times, and *P < 0.05 and **P < 0.01 were considered as statistical significance. [file 12935_2021_2104_MOESM1_ESM.jpg]

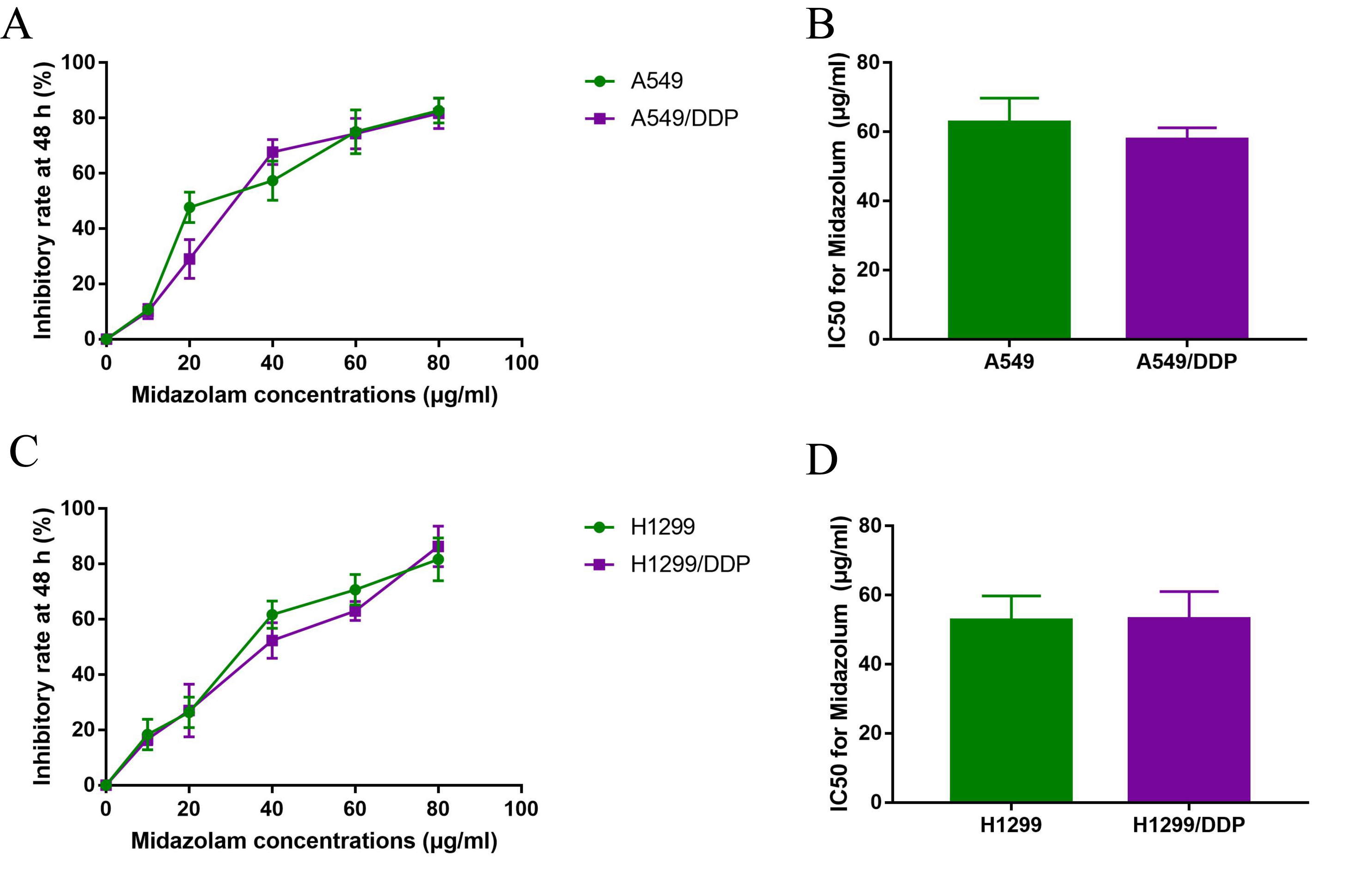

Supplement: Supplementary file 2 — Additional file 2: Figure S2. (A, C) The inhibitory effects of differential doses of midazolam on NSCLC cells. (B, D) The IC50 values for midazolam in CS-NSCLC cells and CR-NSCLC cells were shown. Each experiment repeated at least 3 times, and *P < 0.05 and **P < 0.01 were considered as statistical significance. [file 12935_2021_2104_MOESM2_ESM.jpg]

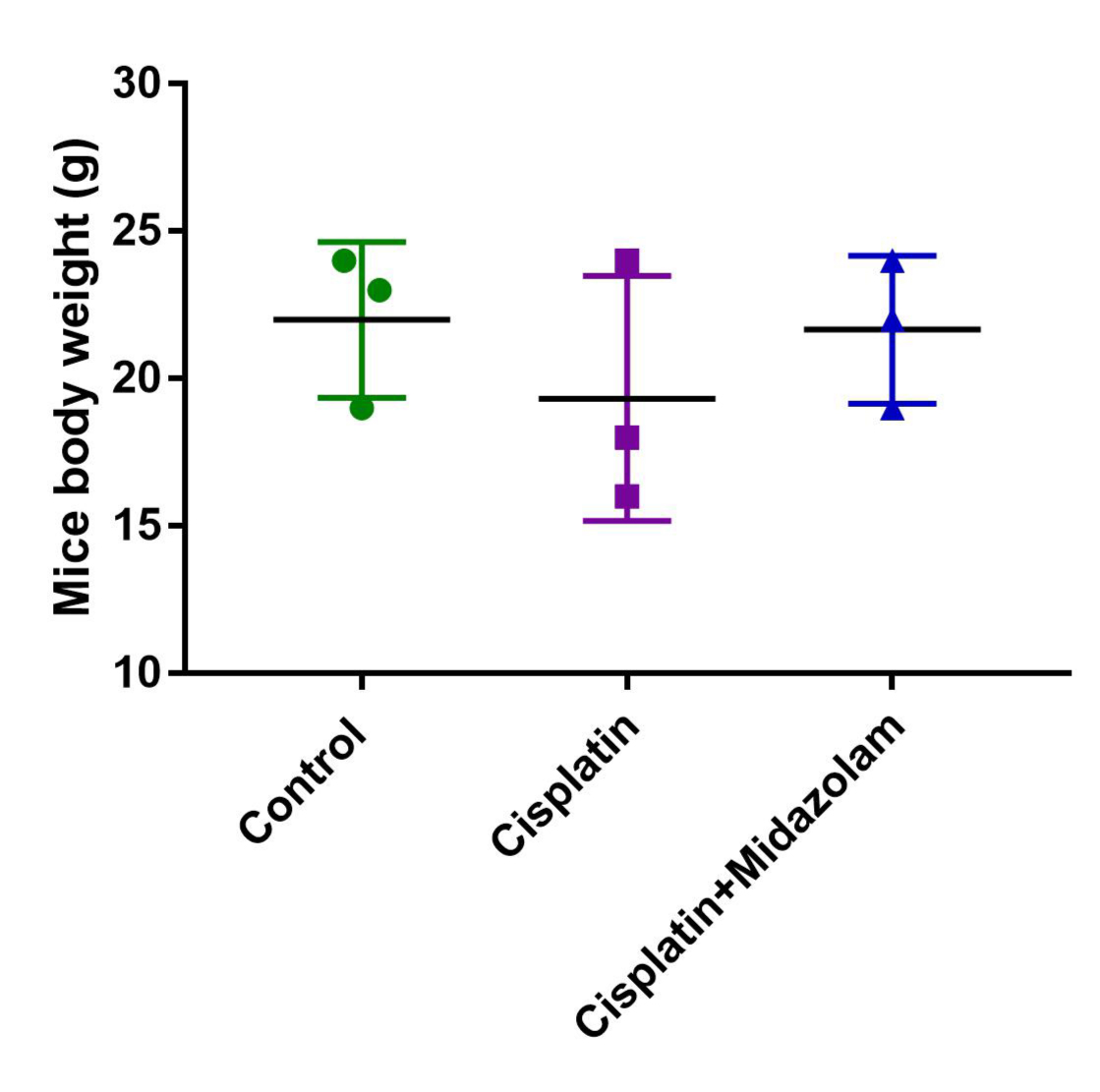

Supplement: Supplementary file 3 — Additional file 3: Figure S3. The mice body weights were measured before surgical resection. Each experiment repeated at least 3 times. [file 12935_2021_2104_MOESM3_ESM.jpg]
